# Reductive Activation of White Phosphorus to [P_4_]^2–^, [P_2_]^2–^, and a Formal P^2–^ Radical by Rare‐Earth Dinitrogen Complexes

**DOI:** 10.1002/anie.6643873

**Published:** 2026-06-21

**Authors:** Arpan Mondal, Richard A. Layfield

**Affiliations:** ^1^ Department of Chemistry School of Life Sciences University of Sussex Brighton U.K.

**Keywords:** gadolinium, magnetism, organometallics, phosphorus, yttrium

## Abstract

Activation of white phosphorus (P_4_) to smaller P*_n_* units by rare‐earth compounds remains a synthetic challenge. Here, we show that the rare‐earth dinitrogen complexes [{(Cp^ttt^)_2 _M}_2_(μ‐1,2‐N_2_)] (M = Y, Gd; Cp^ttt^ = 1,2,4‐tri(*tert*‐butyl)cyclopentadienyl), reductively cleave P_4_ to give a homologous series of phosphorus anions of decreasing nuclearity, that is, [{(Cp^ttt^)_2 _M}_2_(μ‐P_4_)] (**1** **_M_**), [{(Cp^ttt^)_2 _M}_2_(μ‐η^2^:η^2^‐P_2_)] (**2** **_M_**), and [{(Cp^ttt^)_2 _M}_2_(μ‐P)] (**3** **_M_**). Structural, spectroscopic, and computational studies show that **1** **_M_** contains a bicyclobutane‐like [P_4_]^2–^ ligand, whereas **2** **_M_** features a side‐on coordinated [P═P]^2–^ ligand, and **3** **_M_** comprises a monatomic P^2–^ ligand formulated as a phosphorus‐centered radical with *S* = 1/2. The EPR spectrum of **3_Y_** confirms hyperfine coupling to ^31^P and ^89^Y, while magnetic measurements on **3_Gd_** reveal strong antiferromagnetic exchange between the Gd^3+^ ions and the radical ligand. Density functional theory supports a three‐center π‐type M–P–M interaction in **3** **_M_**, with the unpaired spin localized primarily on phosphorus and only weak delocalization onto the metal centers. These findings represent progressive fragmentation of P_4_ to diatomic and monatomic phosphorus anions by rare‐earth reagents, thereby extending the chemistry of multiply bonded phosphorus and persistent phosphorus radicals into the rare‐earth series.

## Graphical abstract

Masked divalent yttrium and gadolinium dinitrogen complexes mediate the fragmentation of white phosphorus, converting P_4_ into discrete dimetallic complexes of [P_4_]^2–^ and [P_2_]^2–^ ligands, and an unprecedented monatomic P^2–^ radical ligand. Structural, spectroscopic, magnetic, and DFT studies reveal insight into the bonding between these unusual P*_n_* units and the rare‐earth elements.

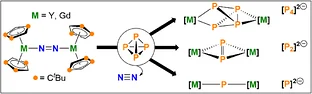

## 1 Introduction

Elemental forms of phosphorus, especially the tetrahedral P_4_ allotrope white phosphorus, are important feedstocks for the industrial synthesis of phosphorus compounds [1, 2, 3, 4, 5]. Part of the attraction of P_4_ as a starting material stems from its strained geometry and reactive nature. Activation of P_4_ by transition metals is well‐established and has provided numerous protocols for the synthesis of phosphorus compounds and novel phosphorus‐containing ligands, thereby circumventing the need to prepare hazardous chlorinated intermediates [6, 7, 8, 9]. Low‐valent main group reagents, including carbenes and silylenes, have also proven to be highly effective at P_4_ activation, delivering a variety of phosphorus compounds of fundamental interest for their chemical bonding properties [10, 11, 12, 13, 14, 15, 16, 17, 18].

Compared to transition‐metal and main‐group systems, activation of P_4_ by rare‐earth compounds is underdeveloped and continues to present a challenge [19, 20, 21, 22]. Nevertheless, low‐valent rare‐earth reagents capable of directly activating P–P bonds offer a promising strategy, with divalent metals, especially classical samarium(II) complexes, featuring prominently [23, 24, 25, 26, 27, 28, 29, 30, 31]. However, P_4_ reduction with such reagents often leads to extended polyphosphides, exemplified by the well‐known [P_7_]^3–^ Zintl‐like anion [32, 33, 34]. These species represent thermodynamic sinks that are difficult to deconstruct or functionalize further. Consequently, it is desirable to achieve the reductive activation of P_4_ using rare‐earth reagents to give smaller and potentially more reactive P*_n_* fragments.

We reasoned that the masked divalent reactivity established for our previously reported dinitrogen complexes of trivalent rare‐earth elements [{(Cp^ttt^)_2 _M}_2_(μ‐1,2‐N_2_)] (M = Y, Tb, Dy, Gd; Cp^ttt^ = 1,2,4‐tri(*tert*‐butyl)cyclopentadienyl) could be extended to the reductive fragmentation of P_4_ [35, 36, 37]. The reactivity concept relies on the electrons stored in the π* orbitals of the [N_2_]^2–^ ligand, which can be transferred to a substrate upon coordination to the metals. Subsequent elimination of N_2_ as a traceless leaving group leaves the reduced substrate bound to the metals, as demonstrated with other rare‐earth dinitrogen complexes [38, 39, 40, 41]. With this approach, reducing reactivity is achieved for trivalent rare‐earth metals, such as yttrium and gadolinium, which do not normally adopt the exotic divalent oxidation state.

## 2 Results

### 2.1 Synthesis and Structural Characterization

**SCHEME 1:**
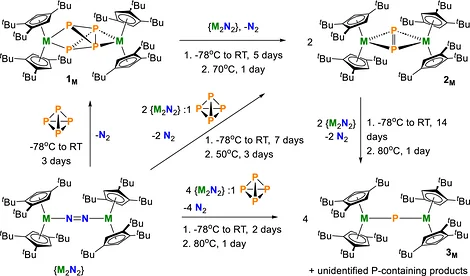
Synthesis of **1** **_M_** (yields: M = Y, 61%; M = Gd, 28%), **2** **_M_** (yields: M = Y, 25%; M = Gd, 24%), and **3** **_M_** (yields: M = Y, 5%; M = Gd, 6%) from {M_2_N_2_}.

Adding toluene to a 1:1 mixture of P_4_ and [{(Cp^ttt^)_2 _M}_2_(μ‐1,2‐N_2_)] (M = Y or Gd) at –78°C (to minimize thermal decomposition of the dinitrogen complexes), followed by warming to ambient temperature and stirring for three days, produced dimetallic [{(Cp^ttt^)_2 _M}_2_(μ‐P_4_)] (**1** **_M_**), with κ^2^:κ^2^‐bridging [P_4_]^2–^ ligands. The isolated yields were 61% (**1_Y_**) and 28% (**1_Gd_**), respectively (Scheme 1). The targeted synthesis of a [P_2_]^2–^ complex was achieved by reacting [{(Cp^ttt^)_2 _M}_2_(μ‐1,2‐N_2_)] with P_4_ in a stoichiometry of 2:1 in toluene, initially at –78°C and then over seven days at ambient temperature followed by heating in THF at 50°C for three days. The resulting dimetallic complexes [{(Cp^ttt^)_2 _M}_2_(μ‐η^2^:η^2^‐P_2_)] (**2** **_M_**), with side‐on bridging [P_2_]^2–^ ligands, were isolated in yields of 25% and 24%, for yttrium and gadolinium, respectively. The attempted synthesis of a μ‐phosphido (P^3–^) ligand by reacting P_4_ with excess amounts of [{(Cp^ttt^)_2 _M}_2_(μ‐1,2‐N_2_)] led only to the isolation of [{(Cp^ttt^)_2 _M}_2_(μ‐P)] (**3** **_M_**), containing a formal P^2–^ ligand, in low yield. Reacting [{(Cp^ttt^)_2 _M}_2_(μ‐1,2‐N_2_)] with P_4_ in the observed 4:1 ratio in toluene at –78°C followed by stirring at ambient temperature for two days and then heating at 80°C overnight generated **3** **_M_** in isolated yields of 5%–6%, with the reaction predominantly generating unidentifiable phosphorus‐containing products (see below). In situ reaction monitoring using ^1^H and ^31^P{^1^H} NMR spectroscopy revealed that **2_Y_** can also be obtained from the reaction of **1_Y_** with the dinitrogen complex, and **3_Y_** can be synthesized by reacting the dinitrogen complex with **2_Y_** in the appropriate stoichiometry (Scheme 1, Figures S16–S19).

**FIGURE 1:**
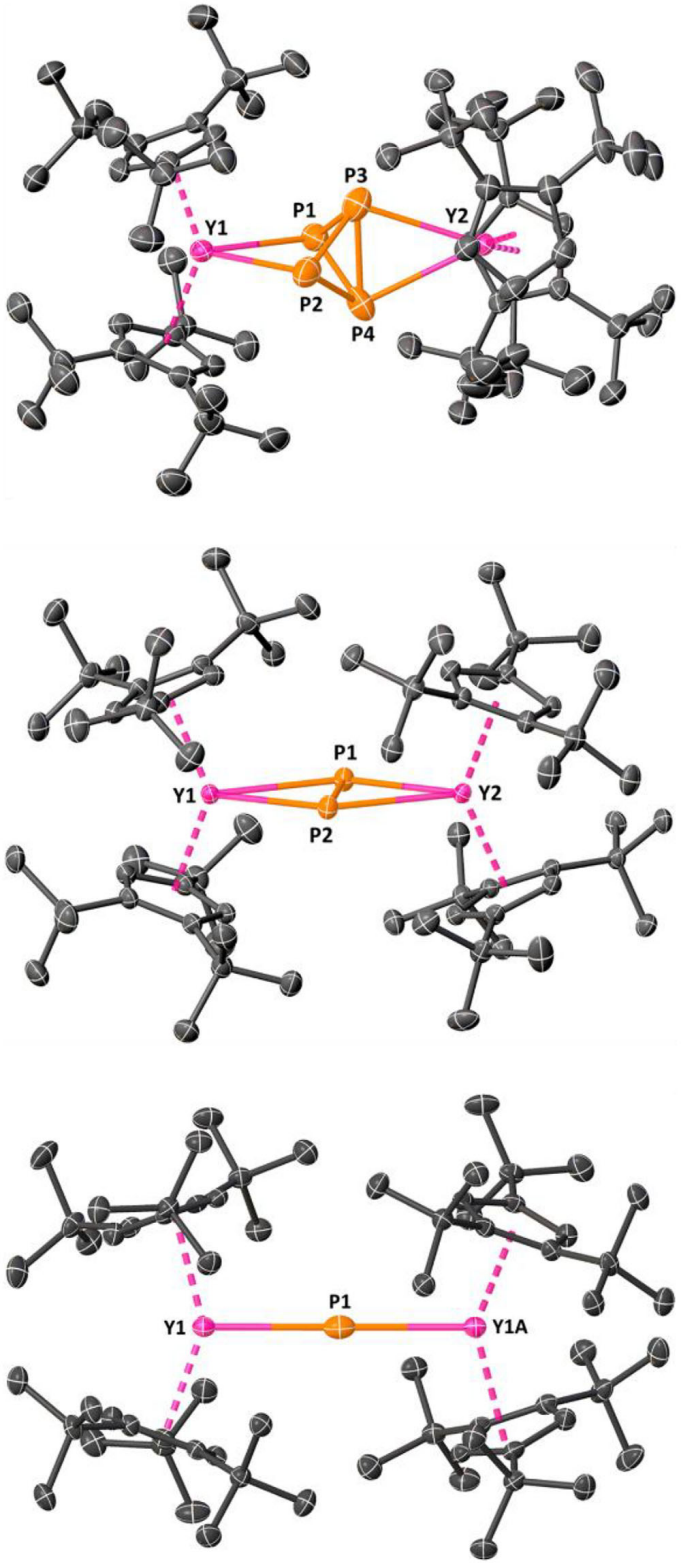
Structures of **1_Y_** (top), **2_Y_** (middle), and **3_Y_** (bottom). Thermal ellipsoids are at the 30% probability level. For clarity, hydrogen atoms are omitted.

The molecular structures of **1** **_M_**, **2** **_M_**, and **3** **_M_** were determined by x‐ray crystallography [42]. The yttrium‐phosphorus complexes are described in detail here (Figure 1), and the isostructural gadolinium complexes are summarized in the Supporting Information (Figures S1–S3, Tables S1–S6). Molecules of **1_Y_** consist of two mutually perpendicular {(Cp^ttt^)_2_Y} units bridged by a [P_4_]^2–^ ligand, which adopts a bicyclobutane structure by virtue of reductive ring opening of P_4_ along one P–P bond. The range of P–P bond lengths in **1_Y_** is 2.158(3)–2.266(3) Å (average 2.211 Å). The [P_4_]^2–^ ligand adopts an unsymmetrical bonding mode in the solid state, with κ^2^‐type coordination of the formally anionic phosphorus atoms producing Y1–P1 and Y1–P2 distances of 2.8473(16) and 2.9142(19), respectively. Side‐on coordination of formally neutral P3 and P4 is also observed, resulting in slightly longer Y2‐P3 and Y2‐P4 distances of 2.9395(18) and 3.0397(17) Å, respectively.

The ^1^H NMR spectrum of **1_Y_** in benzene‐D_6_ at 25°C (Figure S7) consists of two resonances for the cyclopentadienyl protons (6.60, 6.66 ppm) and three for the *tert*‐butyl groups (1.40, 1.67, 1.75 ppm), consistent with their inequivalence in the solid‐state structure and similar to the behaviour reported for other bimetallic rare‐earth metallocenes bearing bulky cyclopentadienyl ligands [43]. The solution‐state behaviour of **1_Y_** was then investigated by variable‐temperature ^31^P{^1^H} NMR spectroscopy. At 25 °C, the spectrum displays a binomial triplet centred on –191.3 ppm due to coupling with ^89^Y (*I* = 1/2, 100%, ^1^ *J* = 232 Hz) (Figure S8), which remains essentially unchanged upon cooling to –60 °C (Figure S9). This observation indicates that all four phosphorus atoms of the [P_4_]^2–^ unit are magnetically equivalent on the NMR timescale over this temperature range, consistent with rapid averaging in solution.

The structure of **2_Y_** features a planar {Y_2_P_2_} core around which the two {(Cp^ttt^)_2_Y} units adopt an eclipsed orientation to facilitate side‐on binding of the [P_2_]^2–^ ligand (Figure 1, Table S4), similar to that often found in rare‐earth and actinide complexes of side‐on [E_2_]^2–^ ligands (E = N, Sb, Bi) [44, 45, 46, 47, 48, 49, 50, 51, 52, 53, 54, 55, 56, 57, 58, 59, 60, 61]. Compared to **1_Y_**, a substantial increase in the Y–P distances is found in **2_Y_**, which lie in the range 2.897(2)–2.916(2) Å. The much shorter distance between the phosphorus atoms of 2.0387(6) Å in **2_Y_** compared to **1_Y_** is indicative of a P═P double bond. Consistent with this, the Raman spectrum of **2_Y_** shows a dominant absorption at 597 cm^−1^ for the [P_2_]^2–^ ligand (Figure S20), with the equivalent absorption at 591 cm^−1^ for **2_Gd_** (Figure S21). These results are comparable to stretching vibrations reported for side‐on [P_2_] complexes of transition metals and uranium [62, 63, 64]. As expected, the P═P stretching vibrations identified for **2** **_M_** occur at much lower energies than those found for rare‐earth complexes of side‐on [N_2_]^2–^ ligands, which are typically in the range 1400–1470 cm^−1^ [65]. Similar to **1_Y_**, the ^1^H NMR spectrum in THF‐D_8_ at 25°C shows two resonances for the cyclopentadienyl protons (5.99, 6.60 ppm) and three for the *tert*‐butyl groups (1.27, 1.36, 1.55 ppm) (Figure S10). The ^31^P{^1^H} NMR spectrum of **2_Y_** features a triplet at 1078.3 ppm, with a small ^31^P‐^89^Y coupling constant of ^1^ *J* = 25 Hz (Figure S11). The markedly different ^31^P chemical shift in **2_Y_** compared to **1_Y_** reflects deshielding of the phosphorus nuclei due to π‐bonding within the [P_2_]^2–^ ligand, which is also consistent with the smaller ^1^ *J*‐coupling of the ^31^P nuclei to ^89^Y.

In the structure of **3_Y_**, the two eclipsed {(Cp^ttt^)_2_Y} units are bridged by a single phosphorus atom and are related by a crystallographic two‐fold rotation axis, which coincides with the bridging phosphorus atom (Figure 1). The resulting Y–P distance is 2.7694(3) Å and the Y1‐P‐Y1A angle is 179.02(6) Å. Based on formal oxidation states of +3 for yttrium and gadolinium in **3** **_M_**, charge balance requires a radical P^2–^ ligand. Whereas phosphido ligands P^3–^ are uncommon in rare‐earth chemistry [66, 67], P^2–^ ligands are, to the best of our knowledge, unknown for any metal. Whilst the bridging ligand in **3_Y_** and **3_Gd_** could be the dianionic phosphido species [PH]^2–^, the IR spectra of these compounds do not show any evidence for a P–H stretch (Figure S6). The ^1^H NMR spectrum of **3_Y_** (Figure S12) does not show a 1:1 doublet indicative of a {PH} unit, consisting instead of singlets for the cyclopentadienyl protons (6.46 ppm) and the *tert*‐butyl groups (1.54, 1.64 ppm). Furthermore, the ^31^P{^1^H} and ^31^P NMR spectra of **3_Y_** both show a triplet at 12.9 ppm and 13.2 ppm, respectively, with a ^31^P‐^89^Y coupling constant of ^1^ *J* = 94.5 Hz and no evidence of ^31^P‐^1^H coupling (Figures S13, S14). The ^31^P{^1^H} NMR spectrum of the oil‐like residue recovered after isolating crystalline **3_Y,_** also provides insight into the low yield of this compound (Figure S15). The spectrum shows a mixture of uncrystallized **3_Y_** and several unidentifiable phosphorus‐containing products, and all attempts to improve the isolated yield of this compound resulted in co‐precipitation of these compounds.

### 2.2 Electronic Structure and Magnetic Properties

**FIGURE 2:**
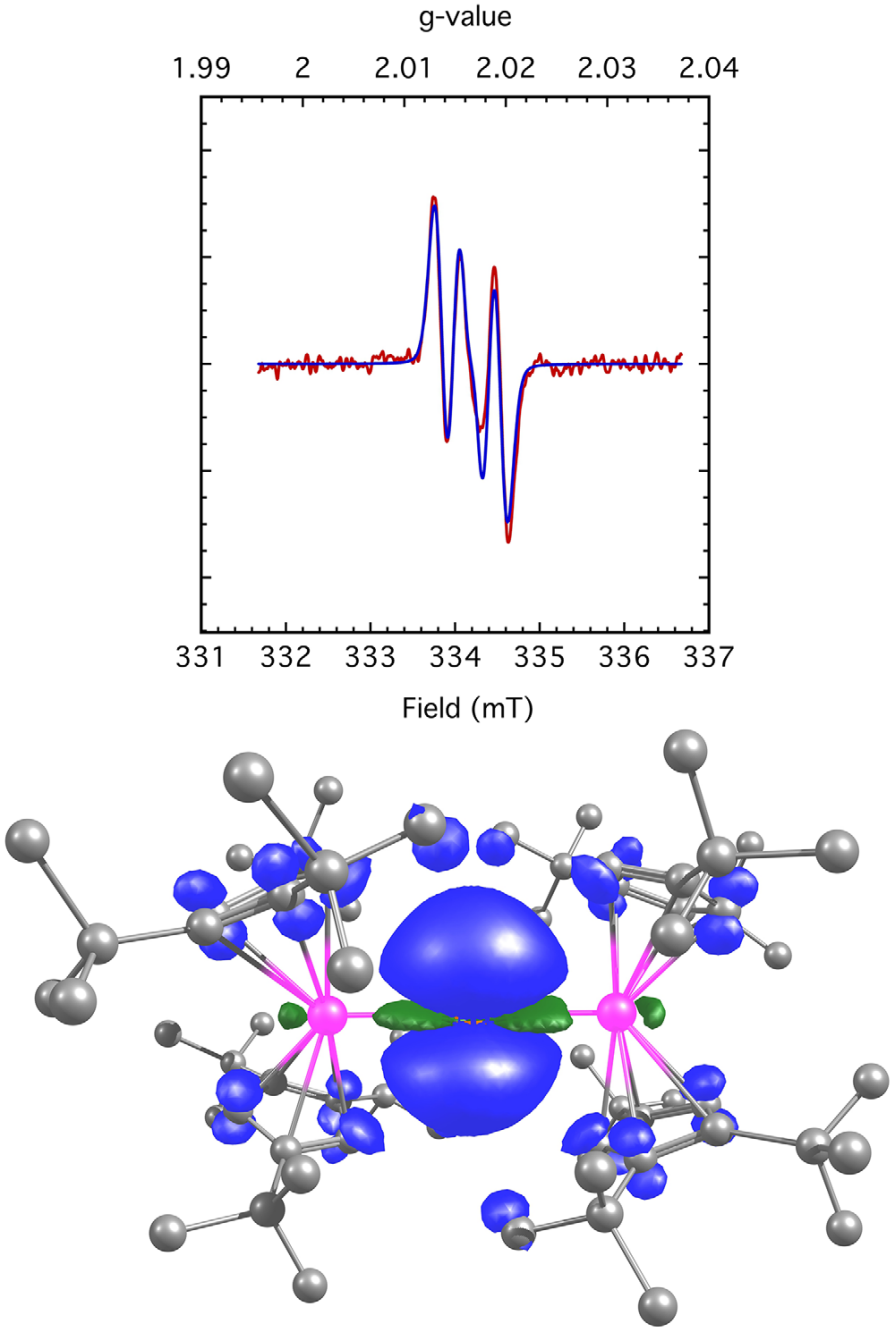
Top: X‐Band EPR spectrum (red) of **3_Y_** in the solid‐state at 100 K. The simulated spectrum in blue used *g* = 2.0097, *A*(^31^P) = 11.9 MHz with lwpp = 0.20 mT, and *A*(^89^Y) = 7.3 MHz with lwpp = 0.045 mT. Bottom: Spin density for **3_Y_** calculated at the B3LYP/def2‐TZVP/def2‐SVP level (isovalue = 0.0006 a.u.).

Considering that a notional P^2–^ ligand would be paramagnetic, **3_Y_** should be EPR active. Thus, the X‐band EPR spectrum of **3_Y_** in the solid state at 100 K features a multiplet centred on *g* = 2.0097 (Figure 2). A simulation of the spectrum was achieved by including small hyperfine coupling of the unpaired spin to the ^31^P (*A* = 11.9 MHz) and ^89^Y (*A* = 7.3 MHz) nuclei. Additional support for the paramagnetic nature of **3_Y_** is provided by DFT calculations of the spin density (Figure 2, Table S7), which show significant accumulation of unpaired spin in a phosphorus 3p orbital, consistent with the small hyperfine coupling, with minor spin polarization on yttrium. Similar calculations on **3_Gd_** reveal a comparable spin density on the bridging phosphorus ligand, in addition to the unpaired spin of the seven 4f electrons on each Gd^3+^ ion (Figure S28, Table S7).

**FIGURE 3:**
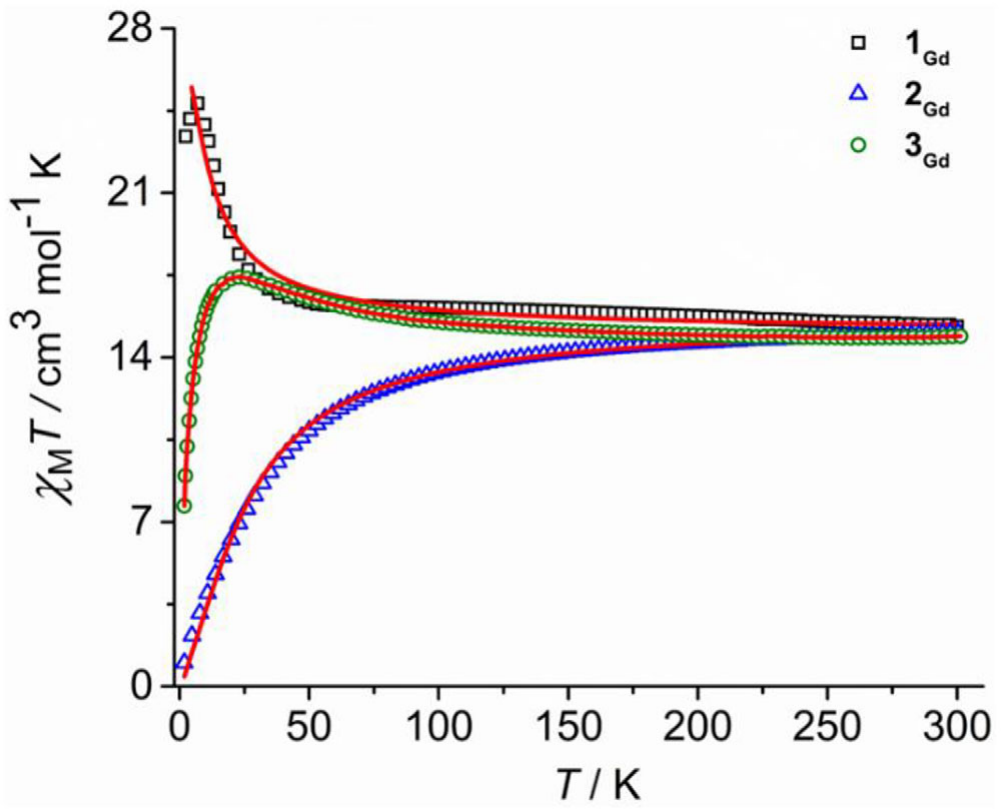
Plots of *χ*_M_ *T*(*T*) for **1_Gd_** (black squares), **2_Gd_** (blue triangles), and **3_Gd_** (green circles) in an applied field of 1000 Oe. Red lines represent fits to the data using the parameters stated in the text.

The radical nature of the bridging phosphorus ligand is also evident in **3_Gd_** from the temperature dependence of the molar magnetic susceptibility (*χ*_M_) in an applied field of 1000 Oe (Figures 3, S26–S28). For **3_Gd_** at 300 K, *χ*_M_ *T* takes a value of 15.10 cm^3^ K mol^−1^ before increasing gradually to reach a maximum of 17.51 cm^3^ K mol^−1^ at 20 K and then decreasing rapidly to 7.69 cm^3^ K mol^−1^ at 2 K, indicating dominant antiferromagnetic exchange. A good fit of the *χ*_M_ *T*(*T*) data was achieved using an isotropic –2*J* spin Hamiltonian containing terms for gadolinium‐radical exchange, gadolinium‐gadolinium exchange, and the Zeeman interaction (Equation S2). Using *g* = 2.02, the fit revealed a strong antiferromagnetic interaction between the Gd^3+^ centres (*S* = 7/2) and a P^2–^ radical (*S* = 1/2) with *J*_Gd‐rad_ = –28.1 cm^−1^, and a non‐negligible antiferromagnetic interaction between the Gd^3+^ centres with *J*_Gd‐Gd_ = –1.7 cm^−1^. The exchange coupling constant determined for **3_Gd_** is very large for a lanthanide, and comparable in magnitude to those found in dimetallic complexes of other organic radical ligands [68, 69, 70].

The magnetic susceptibility properties of **1_Gd_** and **2_Gd_** contrast to those of **3_Gd_** and were modelled using a –2*J* spin Hamiltonian with terms for gadolinium‐gadolinium exchange and the Zeeman interaction (Equation S1). For **1_Gd_**, *χ*_M_ *T* is 15.34 cm^3^ K mol^−1^ at 300 K, rising to 23.32 cm^3^ K mol^−1^ at 2 K. A good fit of the data was achieved with *g* = 1.99 and *J*_Gd‐Gd_ = +0.40 cm^−1^, indicating weak ferromagnetic exchange between the gadolinium centers. In the case of **2_Gd_**, *χ*_M_ *T* is 15.02 cm^3^ K mol^−1^ at 300 K and decreases gradually to 1.04 cm^3^ K mol^−1^ at 2 K. A fit of the data using the isotropic Hamiltonian in Equation S1 with *g* = 2.00 revealed weak antiferromagnetic exchange with *J*_Gd‐Gd_ = –1.02 cm^−1^, similar to that observed in gadolinium coordination complexes with *N*‐ and *O*‐donor ligands [71, 72].

### 2.3 Bonding Analysis

Analysis of the frontier molecular orbitals (MOs) in **1** **_M_**, **2** **_M_**, and **3** **_M_** was undertaken using density functional theory (DFT) calculations. All calculations were performed on the coordinates obtained from the x‐ray structures to reflect accurately the experimental structures (with optimization of the hydrogen atom positions) using the ORCA 6.0.0 software package [73]. The MO analysis gives qualitatively similar results for the yttrium and gadolinium complexes (Figures S30, S32, and S34), hence only the former will be described in detail.

**FIGURE 4:**
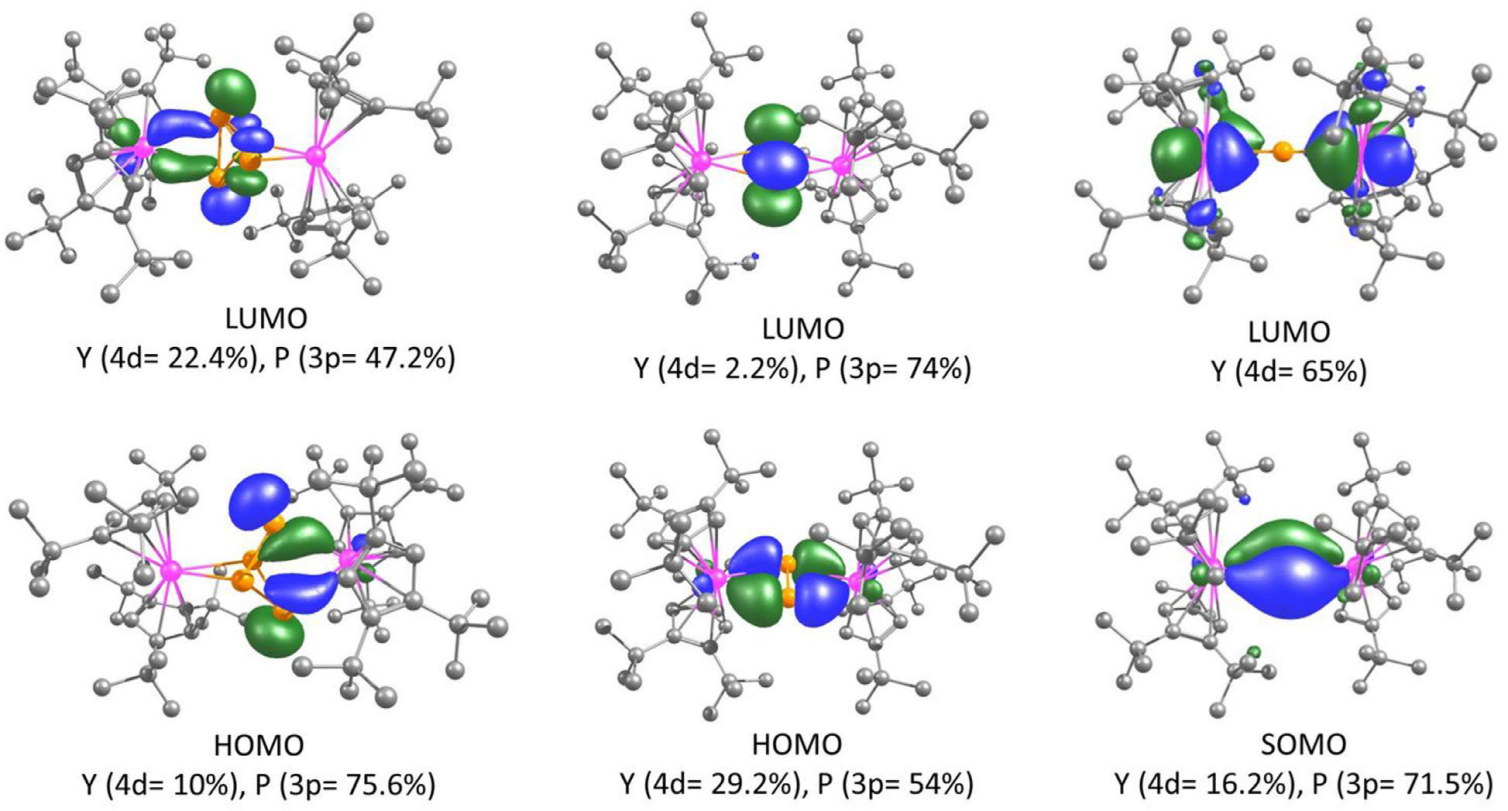
Selected frontier molecular orbitals for **1_Y_** (left), **2_Y_** (center), and **3_Y_** (right). Calculations were performed at the B3LYP/def2‐TZVP/def2‐SVP level (isovalue = 0.033).

In **1_Y_**, the main bonding interaction between yttrium and the [P_4_]^2–^ ligand takes place in the highest‐occupied MO (HOMO) and consists of overlap between the κ‐bound phosphorus 3p orbitals (75.6%) and an yttrium 4d orbital (10%) (Figures 4, S30). The overlap between the other two phosphorus 3p orbitals (corresponding to P3 and P4 in Figure 1) and an yttrium 4d orbital corresponds to the lowest‐unoccupied MO, hence **1_Y_** can be regarded as consisting of an electrostatic interaction between nominal [(Cp^ttt^)_2_Y}(P_4_)]^–^ and [(Cp^ttt^)_2_Y]^+^ fragments.

The analysis of **2_Y_** reveals that the main yttrium‐phosphorus bonding interaction occurs in the HOMO, consisting of overlap between a side‐on [P_2_]^2–^ π* orbital and a 4d orbital on each yttrium atom (Figures 4, S32). A substantial increase in the yttrium contribution to the HOMO is observed in **2_Y_** relative to **1_Y_**, with 29% yttrium 4d character and 54% phosphorus 3p character. The LUMO in **2_Y_** corresponds to the second π* orbital of [P_2_]^2–^ and is perpendicular to the HOMO.

In the radical‐bridged complex **3_Y_**, the singly occupied molecular orbital (SOMO) consists of overlap between a phosphorus 3p orbital (72%) and yttrium 4d orbitals (16%), indicating weak delocalization through a π‐type [Y–P–Y] interaction (Figures 4, S34). The SOMO–1 is a phosphorus‐centered 3p orbital, while the LUMO is mainly yttrium 4d in character. In **3_Gd_**, the analogous interaction shows slightly increased metal contribution (21% 5d) and reduced phosphorus character (66%), consistent with stronger metal–ligand mixing and explaining the strong exchange coupling in this complex (Figure S34).

**FIGURE 5:**
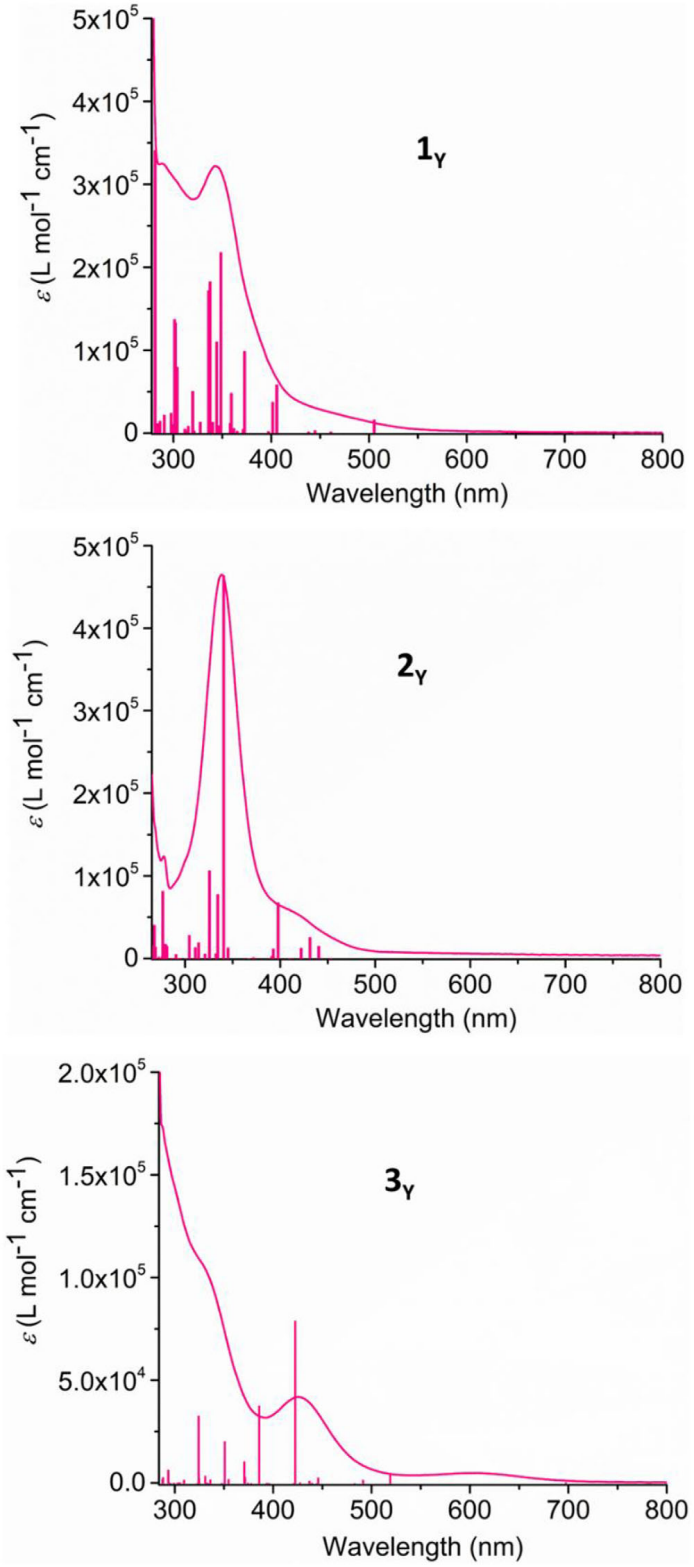
UV–vis spectra of **1_Y_**, **2_Y,_** and **3_Y_** in toluene. Vertical lines are electronic transitions calculated using TD‐DFT at the B3LYP/def2‐TZVP/def2‐SVP level.

Time‐dependent DFT calculations were used to interpret the UV–vis spectra of the yttrium and gadolinium complexes, with the calculated electronic transitions accurately reproducing the experimental spectra (Figures 5, S23–S25, and S42–S44). The spectra of **1_Y_** and **1_Gd_** feature a major absorption centered on *λ*_max_ = 343 and 351 nm, respectively, with no significant absorptions above 450 nm. The TD‐DFT analysis reveals that the main absorption arises from a series of transitions from lower‐lying occupied orbitals, with varying contributions from the metal d‐orbitals and orbitals based on the [Cp^ttt^]^–^ and [P_4_]^2–^ ligands, to unoccupied metal‐centered orbitals (Figures S38, S39, and Tables S8, S9).

The UV–vis spectrum of **2_Y_** consists of a dominant absorption band at *λ*_max_ = 338 nm, which arises primarily from a transition involving the HOMO to the predominantly metal‐centered LUMO+3. A shoulder near 425 nm involves [(Cp^ttt^)_2_Y]^+^ to [P_2_]^2–^ transitions (Figure S40, Table S10). The UV–vis spectrum of **2_Gd_** is similar to that of **2_Y_**, with a main absorption at *λ*_max_ = 340 nm and a poorly defined shoulder absorption around 425 nm (Figures S30, S35, and Table S11). The only feature of note in the UV–vis spectrum of **3_Y_** is a relatively weak absorption at *λ*_max_ = 427 nm, which TD‐DFT shows corresponds to a transition from the [Y–P–Y] bonding MO to yttrium‐centered 4d orbitals (Figure S42, Table S12). The same absorption is poorly defined in the spectrum of **3_Gd_,** but is calculated to occur at 414 nm with appreciable HOMO‐to‐LUMO+1 character (Figure S43, Table S13).

## 3 Discussion

Activation of white phosphorus by rare‐earth complexes under reducing conditions or by nucleophilic addition typically produces polyphosphorus ligands based on networks of P–P single bonds [19, 20, 21, 25, 34]. Similar outcomes are obtained through the activation of transition metal complexes containing polyphosphorus ligands, such as [Cp*Fe(η^5^‐P_5_)] (Cp* = C_5_Me_5_), by low‐valent rare‐earth complexes [23, 26, 27, 28]. Species obtained from these reactions include [P*_n_*]*^x–^* ligands with *n* often seven or eight, and as large as 14 [32, 33]. Reduction of P_4_ by the rare‐earth dinitrogen complexes to give the smaller [P*_n_*]*^x–^* fragments found in **1** **_M_**, **2** **_M_** and **3** **_M_** is a markedly different reaction pathway that highlights the value of the masked divalent synthetic methodology.

To the best of our knowledge, the [P_2_]^2–^ unit in **2** **_M_** has not previously been reported as a ligand in rare‐earth chemistry. Prior to our current study, the only known f‐element complex containing [P_2_]^2–^ is a di‐uranium(IV) species, in which the ligand was generated via a phosphadibenzonor bornadiene P‐atom transfer reagent rather than directly from P_4_ [63]. The formation of **2** **_M_**, despite the greater stability of two P–P σ‐bonds compared with the weaker σ + π combination in [P_2_]^2–^ [74], demonstrates that masked divalent rare‐earth dinitrogen complexes can initiate distinct reactivity relative to classical divalent lanthanides. The fragmentation of P_4_ to give **2** **_M_** also parallels known routes to transition‐metal diphosphorus complexes [7, 8, 75, 76, 77] and complements other approaches to P_2_ ligands, such as the decarbonylation of phosphaethynolate precursors [78, 79].

For **3_Y_** and **3_Gd_**, structural, spectroscopic, magnetic, and computational data support assignment of the monatomic phosphorus ligand as an unprecedented P^2–^ radical with *S* = 1/2, showing only weak [M–P–M] delocalization. The previously reported EPR‐active zirconocene [(Cp*_2_Zr)_2_(μ‐P)] is closely related, but its formulation as a mixed‐valence Zr(III)/Zr(IV) complex with a P^3–^ ligand reflects the accessible higher oxidation state of zirconium [80], which is unfavorable for yttrium and gadolinium. Persistent phosphorus radicals stabilized by main‐group substituents or transition‐metal coordination are well established [81, 82, 83, 84, 85], and the phosphorus‐centered radical character in **3** **_M_** extends this chemistry to the rare‐earth elements, offering new opportunities for designing materials, such as single‐molecule magnets [86]. Furthermore, monatomic phosphorus ligands are extremely uncommon in rare‐earth and actinide chemistry. The only known rare‐earth example is a μ_6_‐P^3–^ ligand in a hexametallic yttrium‐potassium cluster [66, 67], while a handful of thorium(IV) and uranium(IV) analogues feature linear μ‐P^3–^ bridges with bonding arrangements reminiscent of **3** **_M_** [87, 88]. Notwithstanding the low isolated yields of **3** **_M_**, the observation that the dinitrogen complexes react with P_4_ by breaking all six P–P bonds underscores the remarkable reactivity of these bespoke reducing agents.

## 4 Conclusion

Activation of white phosphorus by the masked divalent rare‐earth dinitrogen complexes [{(Cp^ttt^)_2 _M}_2_(μ‐1,2‐N_2_)] provides a unique and systematic route to discrete anionic phosphorus ligands of progressively lower nuclearity. The isolation of [{(Cp^ttt^)_2 _M}_2_(μ‐P_4_)] (**1** **_M_**), [{(Cp^ttt^)_2 _M}_2_(μ‐η^2^:η^2^‐P_2_)] (**2** **_M_**), and [{(Cp^ttt^)_2 _M}_2_(μ‐P)] (**3** **_M_**) (M = Y, Gd) demonstrates complete P–P bond scission by rare‐earth reagents that can be regarded as operating in a masked divalent format rather than by classical divalent lanthanide reduction chemistry. The transformation from [P_4_]^2–^ to [P_2_]^2–^ and finally to the P^2–^ radical highlights the exceptional reducing capacity achievable with this synthetic methodology. The isolation of **2** **_M_** expands the limited nature of the [P_2_]^2–^ ligand in f‐element chemistry to the rare‐earth elements. Furthermore, the synthesis of a monatomic phosphorus ligand directly from P_4_ or from [P_2_]^2–^ is an unusual mode of reactivity. The discovery of a formal P^2–^ radical supported by trivalent rare‐earth metals furnishes a new magnetic building block that supports strong metal‐phosphorus exchange coupling, suggesting new design possibilities for magnetic materials that exploit metal‐radical interactions. The possible applications of **1** **_M_**, **2** **_M_**, and **3** **_M_** as P*_n_* transfer reagents will also be explored in our future work.

## Author Contributions

**Arpan Mondal**: investigation, writing – review and editing, formal analysis, and methodology. **Richard A. Layfield**: conceptualization, funding acquisition, writing – original draft, methodology, writing – review and editing, formal analysis, data curation, supervision, and resources.

## Conflicts of Interest

The authors declare no conflicts of interest.

## Supporting information



Additional research data supporting this publication are available at DOI: 10.25377/sussex.30601316. The authors have cited additional references within the Supporting Information [35, 36, 89, 90, 91, 92, 93, 94, 95, 96, 97, 98, 99, 100, 101, 102, 103, 104, 105].**Supporting File 1**: anie73279‐sup‐0001‐SuppMat.docx.

**Supporting File 2**: anie73279‐sup‐0002‐DataFile.zip.

## Data Availability

The data that support the findings of this study are openly available in Figshare at https://doi.org/10.25377/sussex.30601316.
