# Supplementary material for: Reductive Activation of White Phosphorus to [P4]2–, [P2]2–, and a Formal P2– Radical by Rare‐Earth Dinitrogen Complexes
Source: Angew Chem Int Ed Engl. 2026 Jun 21;65(35):e6643873. doi: 10.1002/anie.6643873 (PMC13502576; doi:10.1002/anie.6643873)
Supplement: Supplementary file 1 [file ANIE-65-e6643873-s001.docx]

**Reductive Activation of White Phosphorus to [P_4_]^2–^, [P_2_]^2–^, and a Formal P^2–^ Radical by Rare-Earth Dinitrogen Complexes**

Arpan Mondal*^a^* and Richard A Layfield**^a^*

*^a^*	Department of Chemistry, School of Life Sciences, University of Sussex,

Brighton, BN1 9QR, U.K.

E-mail:	[r.layfield@sussex.ac.uk](mailto:r.layfield@sussex.ac.uk)

**Contents**

Synthesis and Measurement Details	S1-S3

X-Ray Crystallography	S4-S12

IR Spectroscopy	S13-S15

NMR Spectroscopy	S16-S23

Raman Spectroscopy	S24-S25

UV-vis Spectroscopy	S26-S28

Magnetic Measurements	S29-S31

Computational Details	S32-S53

References	S54

**Synthesis and Measurement Details**

All reactions were carried out under rigorous anaerobic, anhydrous conditions using argon or nitrogen atmospheres and Schlenk and glove-box techniques. All solvents were refluxed over an appropriate drying agent for a minimum of three days (molten potassium for toluene and Na/K alloy for hexane), and then distilled and degassed via a minimum of three freeze-pump-thaw cycles. Solvents were then stored in ampoules over potassium mirrors. Literature procedures were used to synthesise [{(Cp^ttt^)_2_M}_2_(μ-1,2-N_2_)] (M = Y, Gd).^[89, 90]^ P_4_ was purified by sublimation prior to use. FTIR spectra were recorded on a Bruker Alpha spectrometer with a platinum-diamond ATR module, housed in a glovebox. Raman spectra were measured using a Renishaw inVia confocal Raman microscope using an excitation laser wavelength of 532 nm and 1800 mm^–1^ grating. Laser power was kept below 0.5 mW to reduce heating effects. Measurements used a 20× magnification objective, resulting in a laser spot size of about 1.60 mm. Spectra were baseline corrected and cosmic rays were removed using WiRE software. For the baseline, the software automatically uses an ‘intelligent fitting’, which excludes regions with peaks and fits the rest of the spectrum using a polynomial expression. UV-Visible spectra were recorded using a PerkinElmer LAMBDA 265 spectrophotometer. NMR spectra were recorded at 25°C on a Varian VNMRS 400 spectrometer (^1^H = 399.50 MHz, ^31^P = 161.71 MHz) and referenced to external standards as appropriate (SiMe_4_, 85% H_3_PO_4_). X-band EPR spectra were recorded at the PEPR Facility at Imperial College London. Measurements were made in CW mode on a Bruker EMX spectrometer equipped with a Bruker ER049X SuperX microwave bridge, a Bruker ER4122SHQE resonator, and an Oxford Instruments ITC503 temperature controller.

**Synthesis of [{(Cp^ttt^)_2_Y}_2_(P_4_)] (1_Y_)**

Toluene (8 mL) was added to a mixture of [{(Cp^ttt^)_2_Y}_2_(μ-1,2-N_2_)] (100 mg, 0.087 mmol) and P_4_ (10.8 mg, 0.087 mmol) at –78°C. The suspension was slowly warmed to room temperature and stirred for 72 hours, producing an orange-yellow solution with some precipitate. The solvent was then removed under reduced pressure to yield a solid residue, which was washed multiple times with cold hexane and dried under vacuum. [{(Cp^ttt^)_2_Y}_2_(P_4_)] (**1_Y_**) was obtained as a light orange-yellow solid (65 mg, 61%). Single crystals suitable for X-ray diffraction were grown by the slow evaporation of a concentrated hexane or toluene solution. **Elemental analysis** (%), found (calculated) for C_68_H_116_P_4_Y_2_: C 66.01 (66.11); H 9.43 (9.46). **FTIR** ($\tilde{\upsilon}$/cm^–1^): 3000-2800 (m br), 1486-1459 (d, str.), 1393 (s, s), 1358 (s, s), 1236 (s, s), 1163 (s, s), 1001 (s, s.), 836-814 (m, str.), 683-670 (d, str.). **^1^H NMR (C_6_D_6_, *δ*/ppm):** 1.40 (36H, s, *^t^*Bu), 1.67 (36H, s, *^t^*Bu), 1.75 (36H, s, *^t^*Bu), 6.60 (4H, s, C_5_H_2_^t^Bu_3_), 6.66 (4H, s, C_5_H_2_^t^Bu_3_). **^31^P{^1^H} NMR (C_6_D_6_, *δ*/ppm):** –191.3 (t, *J*_Y-P_ = 232 Hz). Due to the poor solubility of **1_Y_**, we were unable to acquire a ^13^C NMR spectrum.

**Synthesis of [{(Cp^ttt^)_2_Gd}_2_(P_4_)] (1_Gd_)**

Compound **1_Gd_** was synthesised using the procedure described for **1_Y_**, using [{(Cp^ttt^)_2_Gd}_2_(μ-1,2-N_2_)] (100 mg, 0.078 mmol) and P_4_ (9.7 mg, 0.078 mmol), and isolated as a light orange-yellow solid (30 mg, 28%). Crystals suitable for single-crystal X-ray diffraction were grown by slow evaporation of a concentrated hexane solution. **Elemental analysis** (%), found (calculated) for C_68_H_116_P_4_Gd_2_: C 59.47 (59.52); H 8.49 (8.52). **FTIR** ($\tilde{\upsilon}$/cm^–1^): 3000-2800 (m br), 1486-1459 (m, br), 1397 (s, s), 1359 (s, s), 1233 (s, s), 1162 (s, s), 999 (s, s.), 836-814 (m, str.), 683-670 (d, str.).

**Synthesis of [{(Cp^ttt^)_2_Y}_2_(P_2_)] (2_Y_)**

Toluene (8 mL) was added to a mixture of [{(Cp^ttt^)_2_Y}_2_(μ-1,2-N_2_)] (100 mg, 0.087 mmol) and P_4_ (5.4 mg, 0.0435 mmol) at –78 °C. The suspension was slowly warmed to room temperature and stirred for seven days, producing a green-yellow solution with some precipitate. The solution turned green-orange upon stirring for three hours at 70°C. The solvent was then removed under reduced pressure to give a solid residue, which was redissolved in THF and stirred for three days at 50°C. A bright green-yellow solution was obtained with some precipitate. The solvent was removed, and the final product **2_Y_** was isolated as a bright green-yellow solid after washing with cold hexane (26 mg, 25%). Crystals suitable for single-crystal X-ray diffraction were grown by the slow evaporation of a concentrated toluene solution. **Elemental analysis** (%), found (calculated) for C_68_H_116_P_2_Y_2_: C 69.40 (69.60); H 9.76 (9.96). **FTIR** ($\tilde{\upsilon}$/cm^–1^): 3000-2800 (m br), 1489-1458 (m, br), 1356 (s, s), 1237 (s, br), 1165 (d, str.), 1110 (s, br), 1068 (s, w), 1000 (s, s), 822-810 (d, str.), 680-671 (d, str.). **Raman** ($\tilde{\upsilon}$/cm^–1^): 596 cm^-1^ (P_2_ stretch).

**^1^H NMR (THF-D_8_, *δ* /ppm):** 1.27 (36H, s, *^t^*Bu), 1.36 (36H, s, *^t^*Bu), 1.55 (36H, s, *^t^*Bu), 5.99, 6.60 (2 × 4H, C_5_H_2_^t^Bu_3_). **^31^P{^1^H} NMR (THF-D_8_, *δ*/ppm):** 1078.3 (t, *J*_Y-P_ = 25 Hz). Due to the poor solubility of **2_Y_**, we were unable to acquire a ^13^C NMR spectrum.

**Synthesis of [{(Cp^ttt^)_2_Gd}_2_(P_2_)] (2_Gd_)**

Compound **2_Gd_** was synthesised using the procedure described for **2_Y_**, using [{(Cp^ttt^)_2_Gd}_2_(μ-1,2-N_2_)] (100 mg, 0.078 mmol) and P_4_ (4.8 mg, 0.039 mmol), and isolated as a bright yellow solid (25 mg, 24%). Crystals suitable for single-crystal X-ray diffraction were grown by slow evaporation of a concentrated toluene solution. **Elemental analysis** (%), found (calculated) for C_68_H_116_P_2_Gd_2_: C 62.35 (62.34); H 8.87 (8.92). **FTIR** ($\tilde{\upsilon}$/cm^–1^): 3000-2800 (m br), 1489-1458 (m, br), 1358 (s, s), 1238 (s, br), 1166 (d, str.), 1109 (s, br), 1069 (s, w), 1000 (s, s), 823-808 (d, str.), 682-670 (d, str.). **Raman** ($\tilde{\upsilon}$/cm^–1^): 591 cm^-1^ (P_2_ stretch).

**Synthesis of [{(Cp^ttt^)_2_Y}_2_(P)] (3_Y_)**

Toluene (8 mL) was added to a mixture of [{(Cp^ttt^)_2_Y}_2_(μ-1,2-N_2_)] (100 mg, 0.087 mmol) and P_4_ (5.4 mg, 0.0435 mmol) at –78°C. The suspension was slowly warmed to room temperature and stirred for two days, producing a blue-greenish solution with some precipitate. The resultant solution was stirred at 80°C overnight, and then the solvent was then removed under reduced pressure to yield a solid, to which toluene (8 mL) and [{(Cp^ttt^)_2_Y}_2_(μ-1,2-N_2_)] (100 mg, 0.087 mmol) were added at –78 °C. The resulting suspension was stirred for 2 days while slowly warming to room temperature. Then the resultant solution turned a green-orange colour while stirring overnight at 80°C. After removing the solvent, the solid was dissolved in a minimum amount of hexane, and a green-orange solution was obtained. The final product **3_Y_** was isolated as green-orange crystals from the slow evaporation of a hexane solution (10.5 mg, 5%). **Elemental analysis** (%), found (calculated) for C_68_H_116_PY_2_: C 71.23 (71.69); H 10.19 (10.23). **FTIR** ($\tilde{\upsilon}$/cm^–1^): 3000-2800 (m br), 1488-1454 (m, br), 1355 (s, s), 1239-1232 (d, br), 1163 (s, br), 1113 (s, w), 1000 (s, s) 820-806 (d, str.), 683-674 (d, str.).

**^1^H NMR (C_6_D_6_, *δ* /ppm):** 1.54 (36H, s, *^t^*Bu), 1.64 (72H, s, *^t^*Bu), 6.45 (4H, s, C_5_H_2_*^t^*Bu_3_). **^31^P{^1^H} NMR (C_6_D_6_, *δ*/ppm):** 13.44, 12.86, 12.27 (P, t, *J*_Y-P_ = 94.5 Hz, P). **^31^P NMR (C_6_D_6_, *δ*/ppm):** 13.24 (t, *J*_Y-P_ = 94.5 Hz).

**Synthesis of [{(Cp^ttt^)_2_Gd}_2_(P)] (3_Gd_)**

Compound **3_Gd_** was synthesised using the procedure described for **3_Y_**, using [{(Cp^ttt^)_2_Gd}_2_(μ-1,2-N_2_)] (200 mg, 0.157 mmol) and P_4_ (4.8 mg, 0.039 mmol), and isolated as a brown-orange solid (11.5 mg, 6%). Single crystals suitable for X-ray diffraction were grown by slow evaporation of a concentrated hexane solution. **Elemental analysis** (%), found (calculated) for C_68_H_116_P_2_Gd_2_: C 63.69 (63.85); H 9.10 (9.14). **FTIR** ($\tilde{\upsilon}$/cm^–1^): 3000-2800 (m br), 1489-1457 (m, br), 1357 (s, s), 1237-1231 (d, br), 1164 (s, br), 1110 (s, w), 1000 (s, s), 823-811 (d, str.), 682-673 (d, str.).

**Conversion of 1_Y_ into 2_Y_**

Toluene (4 mL) was added to a mixture of **1_Y_** (32.5 mg, 0.0263 mmol) and [{(Cp^ttt^)_2_Y}_2_(μ-1,2-N_2_)] (30 mg, 0.0263 mmol) at –78 °C. The suspension was slowly warmed to room temperature and stirred for five days, producing a green-yellow solution with some precipitate. The solution turned green-orange upon stirring overnight at 70°C. At ambient temperature, a bright green-yellow solution was obtained with some precipitate. The solvent was then removed under reduced pressure, and toluene-D_8_ was added. The resulting ^1^H and ^31^P{^1^H} NMR spectra of the crude reaction mixture are shown in Figures S16-S17.

**Conversion of 2_Y_ into 3_Y_**

Toluene (4 mL) was added to a mixture of **2_Y_** (30.9 mg, 0.0263 mmol) and [{(Cp^ttt^)_2_Y}_2_(μ-1,2-N_2_)] (30 mg, 0.0263 mmol) at –78 °C. The suspension was slowly warmed to room temperature and stirred for two weeks owing to the poor solubility of both starting materials, producing a blue-green solution with some precipitate. Then the resultant solution turned a green-orange colour with some precipitate while stirring overnight at 80°C. After cooling to ambient temperature, the solvent was then removed under reduced pressure, and benzene-D_6_ was added. The resulting ^1^H and ^31^P{^1^H} NMR spectra of the crude reaction mixture are shown in Figures S18-S19.

**X-Ray Crystallography**

Single-crystal X-ray diffraction measurements for all compounds complexes were carried out on a Bruker D8 Venture Metaljet diffractometer using Ga-K*_α_* radiation (*λ* = 1.34139 Å). Structures were solved in Olex2 with SHELXT using intrinsic phasing and were refined with SHELXL using least squares minimisation.^[91,92]^ Anisotropic thermal parameters were used for non-hydrogen atoms and isotropic parameters for hydrogen atoms. Hydrogen atoms on carbons were added geometrically and refined using a riding model. Solvent masking was used in the refinement for **2_Gd_** (0.5 hexane), **3_Y_** (1.2 hexane), and **3_Gd_** (2 hexane).

**Table S1.** Crystal data and structure refinement parameters for **1_Y_** and **1_Gd_.**

|  | **1_Y_** | **1_Gd_** |
| --- | --- | --- |
| CCDC ref. code | 2494631 | 2494633 |
| Empirical formula | C_68_H_116_Y_2_P_4_ | C_68_H_116_Gd_2_P_4_ |
| Formula weight | 1235.30 | 1371.98 |
| Crystal system | Monoclinic | Monoclinic |
| Space group | *C*2/*c* | *C*2/*c* |
| *a* (Å) | 42.5294(10) | 42.686(3) |
| *b* (Å) | 10.9107(3) | 10.8873(7) |
| *c* (Å) | 29.6531(7) | 29.6882(19) |
| *α* (°) | 90 | 90 |
| *β* (°) | 99.1930(10) | 99.408(3) |
| *γ* (°) | 90 | 90 |
| *V* (Å^3^) | 13583.1(6) | 13611.5(16) |
| *Z* | 8 | 8 |
| *ρ*_calc_ (g cm^-3^) | 1.208 | 1.339 |
| *F* (000) | 5296.0 | 5696.0 |
| Reflections collected | 77093 | 57796 |
| Independent reflections | 12433 | 12251 |
| *R*_int_ (%) | 0.1218 | 0.0959 |
| GOF on *F*^2^ | 1.036 | 1.086 |
| *R*_1_^a^ | 0.0637 | 0.0618 |
| *wR*_2_^b^ | 0.1683 | 0.1729 |

*^a^* *R_1_*[*I>2σ*(*I*)] = ∑||*F_o_*|−|*F_c_* ||/∑|*F_o_*|; *^b^* *wR_2_*[all data] = [∑{*w*(*F_o_*^2^−*F_c_*^2^)^2^}/∑{*w*(*F_o_*^2^)^2^}]^1/2^

**Figure S1.** Thermal ellipsoid representations (30% probability) of **1_Gd_**. Hydrogen atoms are omitted for clarity.

**Table S2.** Selected bond lengths (Å) and angles (°) for **1_Y_** and **1_Gd_**.

|  | **1_Y_** | **1_Gd_** |
| --- | --- | --- |
| M–C | Y1-C1: 2.720(5)  Y1-C2: 2.872(6)  Y1-C3: 2.824(6)  Y1-C4: 2.643(5)  Y1-C5: 2.656(5)  Y1-C18: 2.651(5)  Y1-C19: 2.809(5)  Y1-C20: 2.850(5)  Y1-C21: 2.708(5)  Y1-C22: 2.669(5)  Y2-C35: 2.656(6)  Y2-C36: 2.827(5)  Y2-C37: 2.805(6)  Y2-C38: 2.634(6)  Y2-C39: 2.624(6)  Y2-C52: 2.616(5)  Y2-C53: 2.786(5)  Y2-C54: 2.815(5)  Y2-C55: 2.664(6)  Y2-C56: 2.621(6) | Gd1-C1: 2.747(10)  Gd1-C2: 2.683(9)  Gd1-C3: 2.686(9)  Gd1-C4: 2.855(9)  Gd1-C5: 2.905(9)  Gd1-C18: 2.738(9)  Gd1-C19: 2.710(9)  Gd1-C20: 2.687(9)  Gd1-C21: 2.847(9)  Gd1-C22: 2.895(10)  Gd2-C35: 2.670(8)  Gd2-C36: 2.706(9)  Gd2-C37: 2.843(9)  Gd2-C38: 2.818(8)  Gd2-C39: 2.649(7)  Gd2-C52: 2.690(8)  Gd2-C53: 2.853(9)  Gd2-C54: 2.664(9)  Gd2-C55: 2.672(9)  Gd2-C56: 2.828(9) |
| M–Cp^ttt^_cent_ | 2.4657(4)  2.4563(5)  2.4247(5)  2.4170(5) | 2.5016(5)  2.4966(4)  2.4608(5)  2.4574(4) |
| M–P | Y2-P4: 3.0397(17)  Y2-P3: 2.9395(18)  Y1-P1: 2.8473(16)  Y1-P2: 2.9142(19) | Gd2-P4: 3.054(3)  Gd2-P3: 2.948(3)  Gd1-P1: 2.856(2)  Gd1-P2: 2.915(3) |
| P–P | P1–P3: 2.200(3)  P1–P4: 2.158(3)  P2–P3: 2.266(3)  P2–P4: 2.216(3)  P3–P4: 2.219(3) | P1–P3: 2.206(4)  P1–P4: 2.167(4)  P2–P3: 2.231(4)  P2–P4: 2.219(4)  P3–P4: 2.234(4) |
| Cp^ttt^-M-Cp^ttt^ | 140.90(3)  142.44(3) | 141.006(14)  142.094(14) |

**Table S3.** Crystal data and structure refinement parameters for **2_Y_** and **2_Gd_.**

|  | **2_Y_** | **2_Gd_** |
| --- | --- | --- |
| CCDC ref. code | 2494634 | 2494629 |
| Empirical formula | C_68_P_2_Y_2_H_116_ | C_71_H_123_Gd_2_P_2_ |
| Formula weight | 1173.36 | 1353.13 |
| Crystal system | Triclinic | Triclinic |
| Space group | *P*$\bar{1}$ | *P*$\bar{1}$ |
| *a* (Å) | 10.5520(5) | 10.5791(4) |
| *b* (Å) | 11.8451(6) | 11.7382(4) |
| *c* (Å) | 28.1698(13) | 30.1520(10) |
| *α* (°) | 89.3970(10) | 98.157(2) |
| *β* (°) | 84.9630(10) | 94.704(2) |
| *γ* (°) | 70.6730(10) | 108.506(2) |
| *V* (Å^3^) | 3309.0(3) | 3482.4(2) |
| *Z* | 2 | 2 |
| *ρ*_calc_ (g cm^-3^) | 1.178 | 1.290 |
| *F* (000) | 1264.0 | 1414.0 |
| Reflections collected | 191437 | 35090 |
| Independent reflections | 16249 | 12607 |
| *R*_int_ (%) | 0.0371 | 0.0621 |
| GOF on *F*^2^ | 1.051 | 1.081 |
| *R*_1_^a^ | 0.0281 | 0.0553 |
| *wR*_2_^b^ | 0.0760 | 0.1618 |

*^a^* *R_1_*[*I>2σ*(*I*)] = ∑||*F_o_*|−|*F_c_* ||/∑|*F_o_*|; *^b^* *wR_2_*[all data] = [∑{*w*(*F_o_*^2^−*F_c_*^2^)^2^}/∑{*w*(*F_o_*^2^)^2^}]^1/2^

**Figure S2.** Thermal ellipsoid representations (30% probability) of **2_Gd_**. Hydrogen atoms are omitted for clarity.

**Table S4.** Selected bond lengths (Å) and angles (°) for **2_Y_** and **2_Gd_**.

|  | **2_Y_** | **2_Gd_** |
| --- | --- | --- |
| M–C | Y1-C1: 2.6567(14)  Y1-C2: 2.6775(14)  Y1-C3: 2.6781(14)  Y1-C4: 2.7703(14)  Y1-C5: 2.7563(14)  Y1-C18: 2.6622(14)  Y1-C19: 2.6856(14)  Y1-C20: 2.6889(14)  Y1-C21: 2.7764(14)  Y1-C22: 2.7667(14)  Y2-C35: 2.6578(14)  Y2-C36: 2.7615(14)  Y2-C37: 2.7732(14)  Y2-C38: 2.6777(14)  Y2-C39: 2.6747(14)  Y2-C52: 2.6503(14)  Y2-C53: 2.7529(13)  Y2-C54: 2.7767(13)  Y2-C55: 2.6859(13)  Y2-C56: 2.6759(14) | Gd1-C1: 2.674(7)  Gd1-C2: 2.714(7)  Gd1-C3: 2.724(7)  Gd1-C4: 2.808(6)  Gd1-C5: 2.786(6)  Gd1-C18: 2.690(7)  Gd1-C19: 2.791(7)  Gd1-C20: 2.806(7)  Gd1-C21: 2.710(7)  Gd1-C22: 2.693(7)  Gd2-C35: 2.702(8)  Gd2-C36: 2.821(9)  Gd2-C37: 2.825(8)  Gd2-C38: 2.712(8)  Gd2-C39: 2.726(8)  Gd2-C52: 2.674(8)  Gd2-C53: 2.781(9)  Gd2-C54: 2.815(9)  Gd2-C55: 2.707(9)  Gd2-C56: 2.711(8) |
| M–Cp^ttt^_cent_ | 2.4223(7)  2.4228(7)  2.4309(8)  2.4219(8) | 2.463(3)  2.461(4)  2.478(4)  2.463(3) |
| M–P | Y1–P1: 2.8676(8)  Y1–P2: 2.8795(7)  Y2–P1: 2.8790(8)  Y2–P2: 2.8784(7) | Gd1–P1: 2.897(2)  Gd1–P2: 2.913(2)  Gd2–P1: 2.901(2)  Gd2–P2: 2.916(2) |
| P–P | 2.0387(6) | 2.049(3) |
| ∠Cp^ttt^-M-Cp^ttt^ | 137.11(3)  136.93(3) | 137.19(13)  137.19(10) |

**Table S5.** Crystal data and structure refinement parameters for **3_Y_** and **3_Gd_**.

|  | **3_Y_** | **3_Gd_** |
| --- | --- | --- |
| CCDC ref. code | 2494630 | 2494632 |
| Empirical formula | C_75.2_H_132.8_Y_2_P | C_80_H_144_Gd_2_P |
| Formula weight | 1245.80 | 1451.41 |
| Crystal system | Monoclinic | Monoclinic |
| Space group | *P*2/*n* | *P*2/*n* |
| *a* (Å) | 18.3883(13) | 18.4057(7) |
| *b* (Å) | 10.3572(7) | 10.3922(3) |
| *c* (Å) | 19.6117(14) | 19.6523(7) |
| *α* (°) | 90 | 90 |
| *β* (°) | 110.072(3) | 110.5580(10) |
| *γ* (°) | 90 | 90 |
| *V* (Å^3^) | 3508.2(4) | 3519.6(2) |
| *Z* | 2 | 2 |
| *ρ*_calc_ (g cm^-3^) | 1.179 | 1.370 |
| *F* (000) | 1354.0 | 1534.0 |
| Reflections collected | 68810 | 46228 |
| Independent reflections | 6414 | 6377 |
| *R*_int_ (%) | 0.0356 | 0.0710 |
| GOF on *F*^2^ | 1.109 | 1.064 |
| *R*_1_^a^ | 0.0464 | 0.0549 |
| *wR*_2_^b^ | 0.1309 | 0.1712 |

*^a^* *R_1_*[*I>2σ*(*I*)] = ∑||*F_o_*|−|*F_c_* ||/∑|*F_o_*|; *^b^* *wR_2_*[all data] = [∑{*w*(*F_o_*^2^−*F_c_*^2^)^2^}/∑{*w*(*F_o_*^2^)^2^}]^1/2^

The phosphorus atoms in **3_Y_** and **3_Gd_** are present on a special position with two-fold axis (1/2 – *x*, *y*, 3/2 – *z*).

**Figure S3.** Thermal ellipsoid representations (30% probability) of **3_Gd_**. Hydrogen atoms are omitted for clarity.

**Table S6.** Selected bond lengths (Å) and angles (°) for **3_Y_** and **3_Gd_**.

|  | **3_Y_** | **3_Gd_** |
| --- | --- | --- |
| M–C | Y1-C1: 2.660(3)  Y1-C2: 2.750(2)  Y1-C3: 2.749(2)  Y1-C4: 2.659(3)  Y1-C5: 2.668(2)  Y1-C18: 2.664(3)  Y1-C19: 2.673(2)  Y1-C20: 2.660(3)  Y1-C21: 2.749(2)  Y1-C22: 2.757(2) | Gd1-C1: 2.711(7)  Gd1-C2: 2.807(6)  Gd1-C3: 2.792(7)  Gd1-C4: 2.686(7)  Gd1-C5: 2.689(6)  Gd1-C18: 2.705(7)  Gd1-C19: 2.701(6)  Gd1-C20: 2.707(6)  Gd1-C21: 2.802(6)  Gd1-C22: 2.801(6) |
| M–Cp^ttt^_cent_ | 2.4139(12)  2.4105(10) | 2.457(3)  2.463(2) |
| M–P | 2.7694(3) | 2.7686(3) |
| ∠Cp^ttt^-M-Cp^ttt^ | 140.80(5) | 140.26(11) |

**Figure S4.** Infrared spectra of **1_Y_** (pink) and **1_Gd_** (green).

**Figure S5.** Infrared spectra of **2_Y_** (pink) and **1_Gd_** (green).

**Figure S6.** Infrared spectra of **3_Y_** (pink) and **3_Gd_** (green).

**Figure S7.** ^1^H NMR spectrum of **1_Y_** in C_6_D_6_ at 25°C.

**Figure S8.** ^31^P{^1^H} NMR spectrum of **1_Y_** in C_6_D_6_ at 25°C.

**Figure S9:** ^31^P{^1^H} NMR spectrum of **1_Y_** in toluene-D_8_ at –60°C.

**Figure S10.** ^1^H NMR spectrum of **2_Y_** in THF-D_8_ at 25°C.

**Figure S11.** ^31^P{^1^H} NMR spectrum of **2_Y_** in THF-D_8_ at 25°C.

**Figure S12.** ^1^H NMR spectrum of **3_Y_** in C_6_D_6_ at 25°C.

**Figure S13.** ^31^P{^1^H} NMR spectrum of **3_Y_** in C_6_D_6_ at 25°C.

**Figure S14.** ^31^P NMR spectrum of **3_Y_** in C_6_D_6_ at 25°C.

**Figure S15.** ^31^P{^1^H} NMR spectrum in toluene-D_8_ at 25°C (in segments) of the oily residue separated from **3_Y_**. No other resonances were observed in the region ±1000 ppm.

**Figure S16.** ^1^H NMR spectrum in toluene-D_8_ of the crude reaction mixture obtained from reacting **1_Y_** with [{(Cp^ttt^)_2_Y}_2_(μ-1,2-N_2_)] to give **2_Y_**.

**Figure S17.** ^31^P{^1^H} NMR spectrum in toluene-D_8_ of the crude reaction mixture obtained from reacting **1_Y_** with [{(Cp^ttt^)_2_Y}_2_(μ-1,2-N_2_)] to give **2_Y_**.

**Figure S18.** ^1^H NMR spectrum in benzene-D_6_ of the crude reaction mixture obtained from reacting **2_Y_** with [{(Cp^ttt^)_2_Y}_2_(μ-1,2-N_2_)] to give **3_Y_**.

**Figure S19.** ^31^P{^1^H} NMR spectrum in benzene-D_6_ of the crude reaction mixture obtained from reacting **2_Y_** with [{(Cp^ttt^)_2_Y}_2_(μ-1,2-N_2_)] to give **3_Y_**.

**Figure S20.** Raman spectra of **2_Y_** (ν_p=p_ = 597 cm^-1^). The spectra are normalised and the baseline is corrected.

**Figure S21.** Raman spectra of **2_Gd_** (ν_p=p_ = 591 cm^-1^). The spectra are normalised and the baseline is corrected.

**Figure S22.** UV-vis spectra in toluene for **1_Y_** (pink, *λ*_max_ = 343 nm) and **1_Gd_** (green, *λ*_max_ = 351 nm).

**Figure S23.** UV-vis spectra in toluene for **2_Y_** (pink, *λ*_max_ = 338 nm) and **2_Gd_** (green, *λ*_max_ = 340 nm).

**Figure S24.** UV-vis spectra in toluene for **3_Y_** (pink, *λ*_max_ = 427 nm) and **3_Gd_** (green).

**Magnetic Measurements**

Magnetic measurements were performed using a Quantum Design MPMS3 SQUID magnetometer equipped with a 7 T magnet. Samples were prepared by gently crushing the crystalline materials before transferring them to a 7 mm NMR tube and covering them in eicosane. The tubes were then flame sealed under a static vacuum. The eicosane was melted in a water bath at 40°C to prevent crystallite torquing. Diamagnetic corrections were performed using Pascal’s constants.^[93]^ Fitting of the magnetic data used equations S1 or S2 implemented in PHI.^[94]^

$\hat{H}=-2J_{Gd-Gd}(\hat{S}_{Gd1}\cdot\hat{S}_{Gd2})+g\mu_{B}\left( \hat{S}_{Gd1}+\hat{S}_{Gd2} \right)\cdot B$ (Equation S1)

$\hat{H}=-2J_{Gd-Rad}\left( \hat{S}_{Gd1}\cdot\hat{S}_{\mathrm{rad}}+\hat{S}_{Gd2}\cdot\hat{S}_{\mathrm{rad}} \right) -2J_{Gd-Gd}(\hat{S}_{Gd1}\cdot\hat{S}_{Gd2})$ $+ g\mu_{B}\left( \hat{S}_{Gd1}+\hat{S}_{Gd2}+\hat{S}_{\mathrm{rad}} \right)\cdot B$

(Equation S2)

**Figure S25.**Left: $\chi_{M}T(T)$ for **1_Gd_**. $\chi_{M}T$ is 15.34 cm^3^ K mol^–1^ at 300 K and 23.32 cm^3^ K mol^–1^ at 2 K. Right: $M(H)$ data at 2 K. The magnetization reaches 14.55 Nβ at 7 T and 2 K. The solid red lines represent the best fit using the isotropic Hamiltonian in equation S1 with *g* = 1.99 and *J* = +0.40 cm^–1^.

**Figure S26.** Left: $\chi_{M}T(T)$ for **2_Gd_**. $\chi_{M}T$ is 15.02 cm^3^ K mol^–1^ at 300 K and 1.04 cm^3^ K mol^–1^ at 2 K. Right: $M(H)$ data at 2 K. The magnetization reaches 5.25 Nβ at 7 T and 2 K. The red lines represent the best fit using the isotropic Hamiltonian in equation S1 with *g* = 2.0 and *J* = -1.02 cm^-1^.

**Figure S27.** Left: $\chi_{M}T(T)$ for **3_Gd_**. $\chi_{M}T$ is 15.10 cm^3^ K mol^–1^ at 300 K, 17.51 cm^3^ K mol^–1^ at 20 K and 7.69 cm^3^ K mol^–1^ at 2 K. Right: $M(H)$ data at 2 K. The magnetization reaches 13.31 Nβ at 7 T and 2 K temperature. The red lines represent the isotropic Hamiltonian in equation S2 with *g* = 2.02 and *J*_Gd-rad_ = –28.05 cm^–1^, *J*_Gd-Gd_ = –1.67 cm^–1^.

**Computational Details**

DFT calculations were performed on the coordinates obtained from the X-ray structures using the ORCA 6.0.0 software package.^[95]^ Hydrogen atom positions were optimized at the DFT level using a pure GGA PBE exchange-correlation functional, keeping constant the positions of other atoms.^[96,97]^ We used the B3LYP functional,^[98-100]^ and relativistic effects were included with the Douglas-Kroll-Hess Hamiltonian (DKH), together with the scalar relativistic contracted version of the basis functions def2-QZVP for Gd, def2-TZVP for Y and P, and def-SVP for C and H atoms.^[101,102]^ The SARC/J auxiliary basis set^[103]^ and RIJCOSX approximation^[104, 105]^ with a TightSCF convergence threshold were used throughout the calculations. The TD-DFT calculations were performed with 60 excited states for Y and 140 states for Gd, respectively, with a CPCM implicit solvent model for toluene.^[106, 107]^

**Figure S28.** Spin density plots for **1_Gd_** (left), **2_Gd_** (centre), and **3_Gd_** (right) calculated at the B3LYP/def2-

QZVP/def2-TZVP/def2-SVP level of theory (isovalue = 0.005 a.u.).

**Table S7.** DFT calculated Mulliken spin densities of the selected atoms for **1_Y_**, **1_Gd_**, **2_Gd_** and **3_Gd_**.

|  | **1_Y_** |  | **1_Gd_** | **2_Gd_** | **3_Gd_** |
| --- | --- | --- | --- | --- | --- |
| Y1 | -0.026 | Gd1 | 7.071 | 7.065 | 7.033 |
| Y1A | -0.026 | Gd2 | 7.091 | 7.066 | 7.033 |
| P | 0.938 | P1 | -0.032 | -0.028 | 0.903 |
|  |  | P2 | -0.005 | -0.029 |  |
|  |  | P3 | -0.035 |  |  |
|  |  | P4 | -0.006 |  |  |

**Figure S29.** Selected frontier molecular orbitals and orbital composition for **1_Y_** calculated at the B3LYP/def2-TZVP/def2-SVP level of theory (isovalue = 0.048).

**Figure S30.** Selected frontier molecular orbitals and orbital composition for **1_Gd_** calculated at the B3LYP/def2-

QZVP/def2-TZVP/def2-SVP level of theory (isovalue = 0.048).

**Figure S31.** Selected frontier molecular orbitals and orbital composition for **2_Y_** calculated at the B3LYP/def2-TZVP/def2-SVP level of theory (isovalue = 0.048).

**Figure S32.** Selected frontier molecular orbitals and orbital composition for **2_Gd_** calculated at the B3LYP/def2-

QZVP/def2-TZVP/def2-SVP level of theory (isovalue = 0.048).

**Figure S33.** Selected frontier molecular orbitals and orbital composition for **3_Y_** calculated at the B3LYP/def2-TZVP/def2-SVP level of theory (isovalue = 0.033).

**Figure S34.** Selected frontier molecular orbitals and orbital composition for **3_Gd_** calculated at the B3LYP/def2-

QZVP/def2-TZVP/def2-SVP level of theory (isovalue = 0.033).

**Figure S35.** UV-vis spectrum of **1_Gd_** in toluene. Vertical lines are electronic transitions calculated using TD-DFT at the B3LYP/def2-QZVP/def2-TZVP/def2-SVP level of theory.

**Figure S36.** UV-vis spectrum of **2_Gd_** in toluene. Vertical lines are electronic transitions calculated using TD-DFT at the B3LYP/def2-QZVP/def2-TZVP/def2-SVP level of theory.

**Figure S37.** UV-vis spectrum of **3_Gd_** in toluene. Vertical lines are electronic transitions calculated using TD-DFT at the B3LYP/def2-QZVP/def2-TZVP/def2-SVP level of theory.

**Table S8.** Computed major excitation wavelengths (*λ*) and oscillator strengths (*f*) in length representation for **1_Y_**.

| ***λ* / nm** | ***f*** | **Dominant contribution** | | |
| --- | --- | --- | --- | --- |
|  |  | **Occupied** | **Virtual** | **%Weight** |
| 730 | 0.00005 | 330 (HOMO) | 331 (LUMO) | 99.2 |
| 505 | 0.00471 | 329 (HOMO-1) | 331 (LUMO) | 96.3 |
| 445 | 0.00084 | 330 (HOMO)  330 (HOMO) | 332 (LUMO+1)  333 (LUMO+2) | 45.3  53.5 |
| 406 | 0.01734 | 325 (HOMO-5)  326 (HOMO-4) | 331 (LUMO)  331 (LUMO) | 33.3  54.7 |
| 402 | 0.01110 | 325 (HOMO-5)  326 (HOMO-4) | 331 (LUMO)  331 (LUMO) | 65.2  28.2 |
| 373 | 0.02945 | 329 (HOMO-1)  330 (HOMO) | 332 (LUMO+1)  334 (LUMO+3) | 14.1  76.7 |
| 359 | 0.01427 | 323 (HOMO-7) | 331 (LUMO) | 88.3 |
| 349 | 0.06511 | 329 (HOMO-1) | 333 (LUMO+2) | 85.8 |
| 345 | 0.03285 | 328 (HOMO-2)  328 (HOMO-2) | 333 (LUMO+2)  332 (LUMO+1) | 78.39  17.7 |
| 338 | 0.05455 | 325 (HOMO-5)  321 (HOMO-9) | 332 (LUMO+1)  331 (LUMO) | 75.3  21.2 |
| 320 | 0.01506 | 326 (HOMO-4)  326 (HOMO-4) | 332 (LUMO+1)  333 (LUMO+2) | 46.4  30.2 |
| 301 | 0.04093 | 320 (HOMO-10)  329 (HOMO-1) | 331 (LUMO)  335 (LUMO+4) | 30.7  34.4 |
| 281 | 0.10192 | 330 (HOMO)  328 (HOMO-1) | 339 (LUMO+8)  334 (LUMO+3) | 61.2  13.4 |

**Figure S38.** Selected molecular orbitals for **1_Y_** corresponding to Table S8, calculated at B3LYP/def2-TZVP/def2-SVP level of theory (isovalue = 0.048 a.u.).

**Table S9.** Computed major excitation wavelengths (*λ*) and oscillator strengths (*f*) in length representation for **1_Gd_**.

| ***λ* / nm** | ***f*** | **Dominant contribution** | | |
| --- | --- | --- | --- | --- |
|  |  | **Occupied** | **Virtual** | **%Weight** |
| 718.7 | 0.000005 | 362a (HOMO)  348b (HOMO) | 363a (LUMO)  349b (LUMO) | 63.8  30.1 |
| 636 | 0.00004 | 362a (HOMO)  348b (HOMO) | 363a (LUMO)  349b (LUMO) | 33.4  65.3 |
| 511 | 0.00407 | 360a (HOMO-2)  347b (HOMO-1) | 363a (LUMO)  349b (LUMO) | 37.1  49.4 |
| 446 | 0.00526 | 359a (HOMO-3)  344b (HOMO-4) | 363a (LUMO)  349b (LUMO) | 52.7  14.8 |
| 399 | 0.021150 | 344b (HOMO-4)  360a (HOMO-2) | 349b (LUMO)  365a (LUMO+2) | 36.5  8.8 |
| 387 | 0.013629 | 348b (HOMO)  348b (HOMO)  343b (HOMO-5) | 351b (LUMO+2)  350b (LUMO+1)  349b (LUMO) | 17.1  11.3  10.3 |
| 353 | 0.036001 | 347b (HOMO-1)  347b (HOMO-1)  347b (HOMO-1) | 352b (LUMO+3)  353b (LUMO+4)  351b (LUMO+2) | 34.1  19.6  10.8 |
| 334 | 0.016887 | 346b (HOMO-2)  359a (HOMO-3)  346b (HOMO-2) | 352b (LUMO+3)  364a (LUMO+1)  358b (LUMO+9) | 17.5  11.5  7.8 |
| 309 | 0.0162047 | 343b (HOMO-5)  343b (HOMO-5) | 357b (LUMO+8)  356b (LUMO+7) | 65.1  5.30 |
| 294 | 0.019276 | 348b (HOMO)  348b (HOMO) | 371b (LUMO+22)  368b (LUMO+19) | 17.60  10.9 |

**Figure S39.** Selected molecular orbitals for **1_Gd_** corresponding to Table S9, calculated at B3LYP/def2-QZVP/def2-TZVP/def2-SVP level of theory (isovalue = 0.048 a.u.).

**Table S10.** Computed major excitation wavelengths (*λ*) and oscillator strengths (*f*) in length representation for **2_Y_**.

| ***λ* / nm** | ***f*** | **Dominant contribution** | | |
| --- | --- | --- | --- | --- |
|  |  | **Occupied** | **Virtual** | **%Weight** |
| 806 | 0.000025 | 315 (HOMO) | 316 (LUMO) | 99 |
| 459 | 0.000587 | 314 (HOMO-1)  315 (HOMO) | 316 (LUMO)  317 (LUMO+1) | 65.5  31.2 |
| 437 | 0.021925 | 313 (HOMO-2) | 316 (LUMO) | 97.5 |
| 402 | 0.0576886 | 311 (HOMO-4) | 316 (LUMO) | 95.1 |
| 344 | 0.3934596 | 315 (HOMO) | 319 (LUMO+3) | 80.3 |
| 329 | 0.0906710 | 312 (HOMO-3)  313 (HOMO-2) | 317 (LUMO+1)  318 (LUMO+2) | 65.7  21.4 |
| 307 | 0.0239642 | 314 (HOMO-1) | 319 (LUMO+3) | 81.2 |
| 279 | 0.069423 | 305 (HOMO-10)  309 (HOMO-6) | 316 (LUMO)  318 (LUMO+2) | 61.8  15.2 |
| 270 | 0.0344181 | 310 (HOMO-5)  315 (HOMO) | 319 (LUMO+3)  320 (LUMO+4) | 66.9  15.4 |

**Figure S40.** Selected molecular orbitals for **2_Y_** corresponding to Table S10, calculated at B3LYP/def2-TZVP/def2-SVP level of theory (isovalue = 0.048 a.u.).

**Table S11.** Computed major excitation wavelengths (*λ*) and oscillator strengths (*f*) in length representation for **2_Gd_**.

| ***λ* / nm** | ***f*** | **Dominant contribution** | | |
| --- | --- | --- | --- | --- |
|  |  | **Occupied** | **Virtual** | **%Weight** |
| 844 | 0.0000030 | 347a (HOMO)  333b (HOMO) | 348a (LUMO)  334b (LUMO) | 63.2  35.9 |
| 488 | 0.004982 | 347a (HOMO) | 349a (LUMO+1) | 94.2 |
| 453 | 0.009400 | 331b (HOMO-2)  330b (HOMO-3)  345a (HOMO-2) | 334b (LUMO)  334b (LUMO)  348a (LUMO) | 40.5  27.1  22.1 |
| 439 | 0.009149 | 344a (HOMO-3)  330b (HOMO-3) | 348a (LUMO)  334b (LUMO) | 54.1  36.1 |
| 424 | 0.038649 | 347a (HOMO)  333b (HOMO) | 351a (LUMO+3)  337b (LUMO+3) | 67.2  17.8 |
| 400 | 0.03057 | 342a (HOMO-5) | 348a (LUMO) | 77.6 |
| 374 | 0.032029 | 333b (HOMO)  333b (HOMO) | 337b (LUMO+3)  338b (LUMO+4) | 50.7  22.3 |
| 362 | 0.053126 | 333b (HOMO)  333b (HOMO)  333b (HOMO) | 340b (LUMO+6)  341b (LUMO+7)  351b (LUMO+17) | 28.9  26.3  10.1 |
| 326 | 0.1758730 | 346a (HOMO-1)  333b (HOMO-5) | 351a (LUMO+3)  351b (LUMO+17) | 34.2  35.6 |
| 285 | 0.038145 | 337a (HOMO-10)  323b (HOMO-10) | 348a (LUMO)  334b (LUMO) | 25.0  19.2 |
| 270 | 0.062817 | 328b (HOMO-5)  328b (HOMO-5)  328b (HOMO-5) | 347b (LUMO+13)  346b (LUMO+12)  351b (LUMO+17) | 17.3  8.5  4.3 |

**Figure S41.** Selected molecular orbitals for **2_Gd_** corresponding to Table S11, calculated at B3LYP/def2-QZVP/def2-TZVP/def2-SVP level of theory (isovalue = 0.048 a.u.).

**Table S12.** Computed major excitation wavelengths (*λ*) and oscillator strengths (*f*) in length representation for **3_Y_**.

| ***λ* / nm** | ***f*** | **Dominant contribution** | | |
| --- | --- | --- | --- | --- |
|  |  | **Occupied** | **Virtual** | **%Weight** |
| 697 | 0.00008 | 306b (HOMO-1) | 308b (LUMO) | 98.1 |
| 519 | 0.01410 | 307b (HOMO)  308a (HOMO) | 309b (LUMO+1)  309a (LUMO) | 68.3  28.8 |
| 491 | 0.00572 | 307b (HOMO) | 310b (LUMO+2) | 97.2 |
| 445 | 0.00954 | 308a (HOMO) | 310a (LUMO+1) | 95.2 |
| 422 | 0.27542 | 308a (HOMO)  307b (HOMO) | 309a (LUMO)  309b (LUMO+1) | 64.7  25.1 |
| 385 | 0.131302 | 301b (HOMO-6) | 308b (LUMO) | 98.3 |
| 370 | 0.036854 | 298b (HOMO-9) | 308b (LUMO) | 98.3 |
| 350 | 0.070970 | 307a (HOMO-1)  306b (HOMO-1) | 312a (LUMO+3)  310b (LUMO+2) | 65.2  25.2 |
| 324 | 0.11437 | 306a (HOMO-2)  304b (HOMO-3) | 310a (LUMO+1)  310b (LUMO+2) | 27.4  23.7 |
| 293 | 0.02278 | 301a (HOMO-7)  303b (HOMO-4)  298a (HOMO-10) | 311a (LUMO+2)  309b (LUMO+1)  310a (LUMO+1) | 33.0  18.8  17.8 |

**Figure S42.** Selected molecular orbitals for **3_Y_** corresponding to Table S12, calculated at B3LYP/def2-TZVP/def2-SVP level of theory (isovalue = 0.048 a.u.).

**Table S13.** Computed major excitation wavelengths (*λ*) and oscillator strengths (*f*) in length representation for **3_Gd_**.

| ***λ* / nm** | ***f*** | **Dominant contribution** | | |
| --- | --- | --- | --- | --- |
|  |  | **Occupied** | **Virtual** | **%Weight** |
| 856 | 0.000090 | 324b (HOMO-1) | 326b (LUMO) | 97.1 |
| 492 | 0.02035 | 340a (HOMO)  325b (HOMO) | 341a (LUMO)  327b (LUMO+1) | 68.7  25.2 |
| 477 | 0.006384 | 322b (HOMO-3) | 326b (LUMO) | 98.9 |
| 444 | 0.006207 | 340a (HOMO) | 342a (LUMO+1) | 96.4 |
| 419 | 0.03136 | 319b (HOMO-6)  325b (HOMO) | 326b (LUMO)  327b (LUMO+1) | 31.3  45.9 |
| 414 | 0.223663 | 319b (HOMO-6)  325b (HOMO) | 326b (LUMO)  327b (LUMO+1) | 66.2  22.4 |
| 406 | 0.034421 | 317b (HOMO-8) | 326b (LUMO) | 93.3 |
| 388 | 0.02158 | 325b (HOMO) | 335b (LUMO+9) | 93.1 |
| 374 | 0.02484 | 325b (HOMO) | 342b (LUMO+16) | 91.0 |
| 359 | 0.057242 | 339a (HOMO-1)  325b (HOMO) | 344a (LUMO+3)  343b (LUMO+17) | 63.1  26.8 |
| 350 | 0.07119 | 325b (HOMO)  339a (HOMO-1) | 343b (LUMO+17)  344a (LUMO+3) | 70.5  22.1 |
| 342 | 0.02161 | 324b (HOMO-1)  336a (HOMO-4) | 327b (LUMO+1)  341a (LUMO) | 58.4  31.0 |
| 307 | 0.045680 | 323b (HOMO-2)  322b (HOMO-3) | 329b (LUMO+3)  328b (LUMO+2) | 36.2  35.4 |
| 290 | 0.02629 | 321b (HOMO-4)  320b (HOMO-5) | 327b (LUMO+1)  330b (LUMO+4) | 18.0  13.0 |
| 282 | 0.045879 | 318b (HOMO-7)  331a (HOMO-9)  317b (HOMO-8) | 327b (LUMO+1)  341a (LUMO)  330b (LUMO+4) | 19.3  16.1  11.3 |

**Figure S43.** Selected molecular orbitals for **3_Gd_** corresponding to Table S13, calculated at B3LYP/def2-QZVP/def2-TZVP/def2-SVP level of theory (isovalue = 0.040 a.u.).

**References**

89. A. Mondal, C. G. T. Price, J. Tang, R. A. Layfield, *J. Am. Chem. Soc.* **2023**, *145*, 20121-20131.
90. A. Mondal, C. G. T. Price, A. Steiner, J. Tang, R. A. Layfield, *Inorg. Chem.* **2025**, *64*, 13309-13317.
91. O. V. Dolomanov, L. J. Bourhis, R. J. Gildea, J. A. K. Howard, H. Puschmann, *J. Appl. Cryst.* **2009**, *42*, 339-341.
92. G. Sheldrick, *Acta Cryst.* **2015**, *71*, 3-8.
93. G. A. Bain, J. F. Berry, *J. Chem. Ed.* **2008,** *85*, 532.
94. N. F. Chilton, R. P. Anderson, L. D. Turner, A. Soncini, K. S. Murray, *J. Comput. Chem.* **2013**, *34*, 1164-1175.
95. F. Neese, *Wiley Interdiscip. Rev.: Comput. Mol. Sci*. **2012**, *2*, 73-78.
96. J. P. Perdew, K. Burke, M. Ernzerhof, *Phys. Rev. Lett.* **1997**, *78*, 1396-1396.
97. J. P. Perdew, K. Burke, M. Ernzerhof, *Phys. Rev. Lett.* **1996**, *77*, 3865-3868.
98. A. D. Becke, *J. Chem. Phys.* **1993,** *98*, 5648-5652.
99. C. Lee, W. Yang, R. G. Parr, *Phys. Rev. B* **1998**, *37*, 785-789.
100. P. J. Stephens, F. J. Devlin, C. F. Chabalowski, M. J. Frisch, *J. Phys. Chem.* **1994**, *98*, 11623-11627.
101. D. Aravena, F. Neese, D. A. Pantazis, *J. Chem. Theory Comput.* **2016**, *12*, 1148-1156.
102. J. Chmela, M. E. Harding, *Mol. Phys.* **2018**, *116*, 1523-1538.
103. J. D. Rolfes, F. Neese, D. A. Pantazis, *J. Comput. Chem.* 2020, **41**, 1842–1849.
104. R. Izsák, F. Neese, *J. Chem. Phys.* **2011**, *135*, 144105.
105. F. Neese, F. Wennmohs, A. Hansen, U. Becker, *Chem. Phys.* **2009**, *356*, 98–109.
106. Y. Takano, K. N. Houk, *J. Chem. Theory Comput.* **2005**, *1*, 70-77.
107. M. Garcia-Ratés, F. Neese, *J. Comput. Chem.* **2020**, *41*, 922-939.
